# Validation of the Ambivalent Ageism Scale in China

**DOI:** 10.1093/geroni/igaa057.1886

**Published:** 2020-12-16

**Authors:** Xin Zhang

**Affiliations:** Peking University, Beijing, China

## Abstract

Two studies were conducted to validate the Ambivalent Ageism Scale in China. In the first study, 474 Chinese adults (18-58) were asked to take the Chinese version of the AAS. EFA exhibited a similar factor solution as the original study, with high internal consistency and construct validity. Moreover, in a second study, 372 Chinese adults (18-85) took the AAS and provided their estimations of the similarities between their current and their past/future self via the SIC. Results indicated that all three factors of the SIC positively related to hostile ageism, whereas succession and identity positively related to benevolent ageism and consumption negatively related to it. Additionally, past self-continuity was positively associated with hostile ageism, and future self-continuity was negatively associated with it, but neither form was associated with benevolent ageism. These results further validate the AAS in China and also provide evidence for the uniqueness of benevolent ageism.

